# Diffusion and Kinetic Theory on Very Long Time and Large Space Scales

**DOI:** 10.3390/e26121037

**Published:** 2024-11-30

**Authors:** Christopher Essex, Bjarne Andresen

**Affiliations:** 1 Department of Mathematics, Middlesex College, The University of Western Ontario, London, ON N6A 5B7, Canada; essex@uwo.ca; 2 Niels Bohr Institute, University of Copenhagen, Jagtvej 155 A, DK-2200 Copenhagen N, Denmark

**Keywords:** thermodynamics, large space and time scales, grand regime, epitropy, kinetic theory

## Abstract

This paper extends the concept of epitropy, as introduced in previous work, to capture the effects of extreme tail behavior arising naturally over very long time and large space scales. Epitropy has some qualities that parallel entropy, although it is not quite the same. Its function is to capture the effects of a probability distribution function (PDF) having only a finite populated domain, which was introduced to eliminate divergent moment integrals. Unlike entropy, it represents hidden properties from the *external* (not the internal) that influence other regimes. This paper uses kinetic theory methods to show the necessity of epitropy in order to ensure that divergent moment integrals do not diverge. While on laboratory scales, the issues in question are negligible, we arrive at dynamics for the grand regime where the tail-generated epitropy can drive the movement of energy.

## 1. Introduction

### 1.1. General

This paper develops on the idea of “slow time” laid out in our previous work [1,2,3,4,5,6]. The concept extends from the well-known pair of regimes of the microscopic and the macroscopic regimes as are typical in statistical mechanics and kinetic theory. This pair of regimes are singled out from other known underlying regimes of mass–energy and space–time scales, wherein quantum and relativistic effects must be considered.

Antonino Zichichi attributed to Anderson and Feynman a vision of nature where underlying structures may be ignored [2,7]. The present paper and its predecessors [1,2,3,4,5,6] treat the laboratory regime as the underlying one. Each of these regime pairs, such as the classical microscopic and macroscopic (i.e., laboratory) regimes, have particular physical variables and accompanying mathematical structures or equations that do not appear to hold at both scales. The microscopic regime has the classical dynamics of molecules, while the macroscopic regime has thermodynamics and fluid mechanics. Statistical mechanics and kinetic theory bridge these two regimes.

The slow-time project proposes that a new regime in the hierarchy probably exists that has not yet been considered. Such a regime would be to our human-scaled regime as we are to the microscopic one. Our regime would be relatively microscopic in comparison to it. Accordingly, we revise the traditional terminology of *macroscopic* regime to *laboratory* regime, so as not to suggest that the laboratory regime represents the final stage in the ladder of regimes. The new regime, beyond the laboratory regime, we call the *grand* regime.

Historically, many attempts have been made to go from fluid dynamical equations to flows on some averaged regime, which would be tantamount to aiming for the grand regime. The famous closure problem of turbulence is the classical example [8]. A similar approach was tried by Saltzman [9] for climate; his work spawned the Lorenz equations [9]. All of these approaches failed. No new regime that could “ignore” the laboratory regime emerged.

One might speculate that this failure at describing, e.g., turbulence is due to something lost in the transition from the microscopic to the laboratory regime instead of a failure of vision. Classical fluid dynamics, as deduced from classical kinetic theory, is after all perturbative with discarded higher-order terms. While the discarded components may be small, small things can accumulate and emerge to affect averages (see the virtual butterfly effect [10]). This suggests a direct move from the microscopic to the grand regime is needed to avoid the issue. This can be achieved, similarly to kinetic theory, with moment integrals, but with revised probability distribution functions (PDFs), as have been developed in the previous work of the slow-time program [1,2,3,4,5,6].

### 1.2. Slow Time and Fluctuating Local Equilibrium

Revising PDFs through convolution has been the strategy of our previous works on this topic. We have developed the convolution PDFs by considering fluctuating temperatures and velocities based on various assumptions about local equilibrium in the laboratory regime [1,2,3,4,5,6]. These have led us to discover that some quantities like temperature disappear, and issues over turbulence do not emerge. Accordingly, new variables emerge in capturing the phenomena of the grand regime.

In the first instance, we fluctuated wind in a Gaussian manner in terms of Gaussian precision [3]. Such a choice is plausible but speculative. Nonetheless, if it is so fluctuated, the wind can be thermalized fully. That is, the wind fluctuations act like kinetic movements leading to a revised temperature-like quantity on the grand scale. That is technically distinct from temperature in the laboratory regime.

If we next fluctuate the laboratory regime temperature similarly through a Gaussian or truncated Gaussian convolution, using Gaussian precision, the resulting PDF is not Gaussian at all, but a new, remarkable hybrid with a Gaussian core and heavy or light tails [11]. This hybrid structure arises in part because of the temperature-dependent normalization of the Maxwellian PDF. It is most important to observe that the Maxwellian-like form is essential for the existence of the concept of temperature in the statistical mechanics of classical physics. Non-Gaussian behavior means *no classical temperature exists* in the grand regime.

Gaussian convolution is a plausible guess but is not definitive. Moreover, the temperature-like parameter that emerges is the result of the Gaussian assumption and is not native to the grand regime that it is meant to help characterize. Many dynamical systems have finite attractor set diameters, so an alternative convolution form, the box car function with sharp finite cutoffs, was also explored [11]. In this case, the grand-regime PDF becomes sub-Gaussian in the tails. Once again, the result is not Gaussian, so no classical temperature can arise. The box car function clips the tails so no heavy tails can emerge.

### 1.3. Epitropy and Kinetic Theory

However, there is more to the idea than the loss of classical variables; there is the emergence of new physical variables. In the first instance, we have the generalized temperature-like variable from the thermalization of wind. But much more significant is the emergence of a new variable we termed *epitropy*. It is a new measurable (in principle) quantity that captures the true tail of the probability distribution function. While this quantity was introduced in [6] for the specific case of Gaussian convolutions, it is here extended to capture other cases of convolution PDFs [11]. Epitropy captures the true tail behavior that Maxwellian thinking can falter at because velocity moments can diverge when hybrid PDFs emerge.

A key result of this paper is that epitropy, understood against an ideal classical Gaussian form, permits the appropriate moment integrals for a diffusion equation for energy via kinetic theory-like forms. But energy is now formulated in terms of generalized temperature and epitropy. This implies that even in the absence of generalized temperature gradients, epitropy gradients can cause energy diffusion in the grand regime.

## 2. Super- and Sub-Gaussian PDFs

In our previous paper on slow time [11], we investigated the qualitative difference resulting from the tail behavior of velocity probability distributions p(x). Proceeding in one dimension (three dimensions will be discussed later), the Gaussian form over an infinite domain in *x* is the presumptive PDF, unless there is specific evidence to the contrary.

(1) $$\begin{array}{c} p\left(x\right)=\frac{\psi }{\sqrt{\pi }}\;e^{-\psi^{2}x^{2}}, \end{array}$$

where ψ is the Gaussian precision. In the case of a Maxwellian velocity distribution, $\psi =1/\left(\sqrt{2}\sigma \right)=\sqrt{m/(2kT)}$, with σ being the standard deviation, and x=v−u is the velocity minus the rest velocity *u*. Precision has units of 1/velocity in that case.

Formally, when moving from the laboratory scale to the slow-time (grand) scale, we first convolved the Boltzmann velocity distribution with a Gaussian distribution in velocity fluctuations about zero *u*. While one may conceive of velocity changes over long times averaging to zero, this assumption is not necessary. There can be persistent winds over long timescales, but that will not change our qualitative observations.

The result of this convolution is

(2) $$\begin{array}{c} p\left(v;\theta \right)={\left(\frac{m}{2k\theta }\right)}^{1/2}\frac{1}{\sqrt{\pi }}\;e^{-\frac{m}{2k\theta }v^{2}}, \end{array}$$

which is a Maxwell-like velocity distribution too, but with a revised effective temperature, θ,

(3) $$\begin{array}{c} \theta =T+\frac{\sigma_{u}^{2}m}{k}\;=\frac{\sigma^{2}m}{k}+\frac{\sigma_{u}^{2}m}{k}, \end{array}$$

where $\sigma_{u}$ is the standard deviation of the Gaussian fluctuations in the wind with velocity *u* [3]. This indicates that the effective temperature of the convolved velocity distribution consists of the sum of contributions from the integration to the laboratory scale and the integration to the grand scale. This is true in general as long as the fluctuating quantity appears exclusively in the exponent of the Gaussian distribution. An example where this condition is not satisfied (temperature) follows below.

θ functions like the classical temperature, but it is slightly larger than the common laboratory-scale temperature, *T*. However, it is a completely new object native to the grand scale. We say that wind is fully “thermalized” in this scenario.

Gaussian fluctuating *v* preserves a temperature-like quantity, θ. On the other hand, fluctuating temperature itself, by fluctuating the precision of the velocity distribution, ψ, cannot achieve this, because ψ is also part of the normalization of the PDF in Equation (1). On the grand scale, fluctuated temperatures produce non-Gaussian forms, which preclude the existence of a temperature in any normal sense as the Gaussian form is necessary for a kinetic temperature to exist.

If one does fluctuate ψ by presuming a Gaussian form for the fluctuations, the convolutions result in

(4) $$\begin{array}{c} p_{\mathrm{fat}}\left(v\right)=\frac{w^{3}\psi_{0}}{\sqrt{\pi }{\left(v^{2}+w^{2}\right)}^{3/2}}\;\exp\left(-\frac{w^{2}\psi_{0}^{2}v^{2}}{v^{2}+w^{2}}\right), \end{array}$$

provided one assumes an infinite domain for the fluctuations in ψ, and *w* is the precision of these fluctuations [3]. *w* represents a transition velocity where the Gaussian PDF form turns into polynomial PDF forms. To see this, for large *v*, the prefactor goes as $∼v^{-3}$, while the exponential approaches a constant [3]. $\psi_{0}$ is the reference ψ about which the fluctuation occurs. Equation (4) is super-Gaussian and yields divergent second moments.

Such heavy tails can be avoided if instead we assume a convolution function with limited range, e.g., a normalized box car distribution,

(5) $$\begin{array}{c} f\left(\xi \right)=\left\{\begin{array}{c} 0\;\mathrm{for}\;|\xi |>\frac{1}{\omega }\\ \omega \;\mathrm{for}\;|\xi |\leq \frac{1}{\omega }. \end{array}\right. \end{array}$$

**Figure 1:**
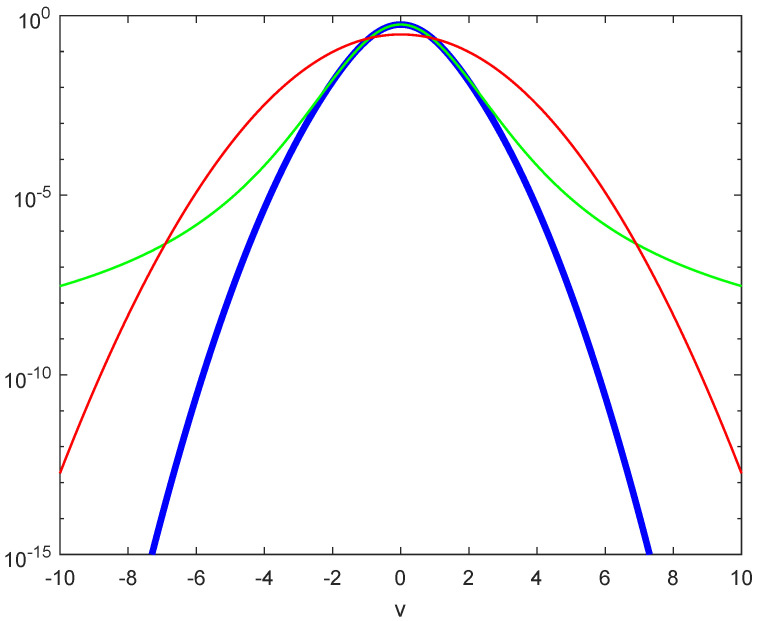
The fat-tail speed distribution in Equation (4) arising from a convolution with Gaussian varying precision is shown in green. The blue curve represents a similar convolution but with a box car PDF, resulting in a sub-Gaussian speed distribution (Equation (6)). A standard Gaussian speed distribution with the same core as the other two is included in red for comparison.

The resulting slow-time distribution,

(6) $$\begin{array}{c} p_{\mathrm{slim}}\left(v\right)=\frac{\omega }{4\sqrt{\pi }}\frac{1}{v^{2}}\left[1-e^{-\frac{4\psi_{0}}{\omega }v^{2}}\right]\;e^{-{\left(\psi_{0}-\frac{1}{\omega }\right)}^{2}v^{2}} \end{array}$$

is sub-Gaussian with its $v^{-2}e^{-v^{2}}$ tail, and thus, second moments will exist. These two convolved tail behaviors are compared with the pure Gaussian $e^{-v^{2}}$ in Figure 1 [11]. All three distributions have the same Gaussian core but differ significantly in the tails.

## 3. Epitropy: Removing Divergence

For the Maxwellian case, there is no concern about second moments because of the exponential decline for large *v*. Thus, generally, one need not consider what happens in the Maxwellian tails of classical kinetic theory, which presumably have negligible effects. However, on the grand scale, temperature fluctuations require consideration of tail behaviors.

There is no a priori way to determine what the correct convolution PDF for fluctuations must be, since we have no experimental evidence for behavior at that scale (yet). The two examples in the preceding section, however, demonstrate that there are two cases to consider: super- and sub-Gaussian. Moreover, if these cases exist in one dimension, the extension to three dimensions is implied in terms of divergence issues.

Physical limitations, e.g., finite energy and finite number, will not allow for divergent integrals despite the divergent cases in the preceding section. The second moment of the Maxwellian expresses total kinetic energy due to the quadratic in $\frac{1}{2}mv^{2}$. We may presume that this is also the case for the grand-regime PDFs. If the latter is divergent, one must conclude that there is something false about the PDF. Moreover, if there is such a problem, it must be rooted in the Maxwellian convolution from which it is derived.

The traditional Maxwellian form avoids such scrutiny because any resulting errors are very small for convergent integrals. However, the Maxwell–Boltzmann *v* distribution presumes a finite number of particles with finite overall energy. This implies a maximum velocity for any particle, as there cannot be more energy for any one particle than exists collectively in the entire ensemble [3]. Beyond the velocity corresponding to that energy, there can only be zero probability for a physical PDF. The simple Maxwellian cannot deliver this property.

**Figure 2:**
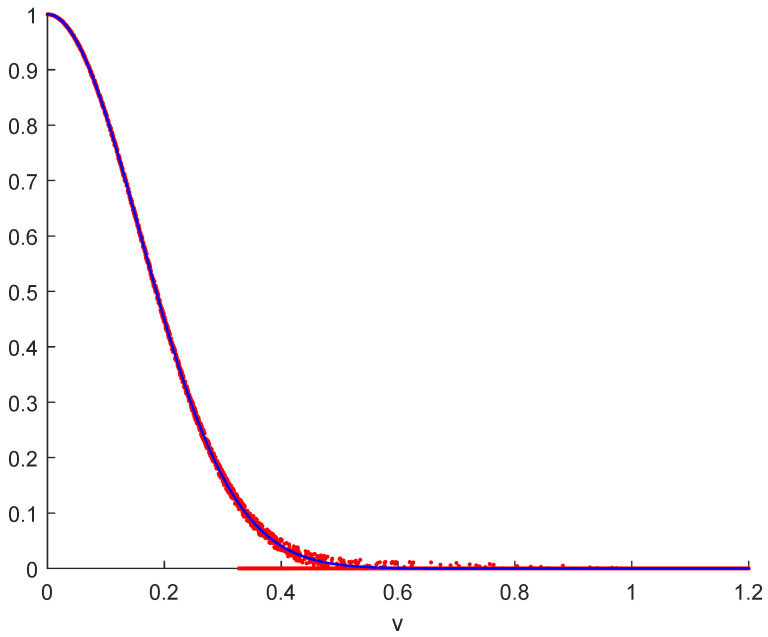
A real Gaussian velocity distribution (red points) will, for physical reasons, drop to zero for velocities greater than when the total energy of the system is concentrated in a single particle (defined here as v=1). However, differences from the continuous mathematical convolution formulation (blue curve) already show up at much lower velocities, about v=0.25. The dots on the *v*-axis illustrate that the probability of having most of the energy of the system concentrated into one or a few particles is so incredibly small that it cannot be physically assessed. Moments in the continuous mathematical formulation diverge, whereas moments in the discrete physical formulation remain finite.

Figure 2 is a cartoon illustration of this situation. In addition to the usual abstract continuous mathematical velocity distribution (blue) extending all the way to infinity, the figure shows the physical velocity distribution (red), which is different on several counts. First of all, any physical system consists of a finite number of discrete particles. Traditionally, that difference between physics and mathematics is glanced over without mention because the moment integrals on the micro- and laboratory scales converge just fine. Not so on the grand scale. The tails really matter, as we will see.

The range of Figure 2 may be split qualitatively into four regions: (i) For low velocities, roughly *v* = 0–0.25, we can without problems ignore velocity bins in any measurement, and the continuum approximation works well and accurately. (ii) For larger velocities, roughly *v* = 0.25–0.6, the continuum approximation begins to break down, measurement bins are no longer of negligible size (horizontal fuzziness), and their populations become stochastic (vertical fuzziness), including no population at all (points on the *v*-axis). (iii) For very large velocities, roughly *v* = 0.6–1, most velocity bins are empty because of the unfavorable combinatorics for focusing so much of the total available energy into a few particles. (iv) The true physical velocity distribution drops to zero for velocities greater than when the total energy of the system is concentrated in a single particle (defined here as v>1).

The mathematical divergence of some convolved distributions mentioned above rests in the very small but infinitely long contributions in the physically non-existent region v>1. This issue cannot practically emerge on laboratory scales [11]. However, divergent convolutions cause it to emerge in the grand regime.

This marginal issue only emerges as tails in the grand regime become significant in the divergent cases. Previously, we attempted to capture this notion specifically in terms of Equation (4) by introducing the concept of *epitropy*. It was originally tied specifically to that particular PDF. However, it is clear that the concept needs to account for the range of possible convolution PDFs that can be in play.

To achieve this, we need to revise the notion of epitropy slightly. The revised version parallels entropy as before. Like entropy, epitropy affects other regimes in a hidden manner. One difference is that epitropy concerns invisible effects on other regimes through the external and not the internal phenomena, namely, how unknown tail behaviors manifest physically. In particular, epitropy arises from unknown convolution integral tails and not from probability considerations like entropy. There is no expectation that epitropy should have relationships to energy through a temperature. Such a relationship between energy, entropy, and temperature exists only in the laboratory regime. In the grand regime, temperature only comes in as a guest that we explicitly invited; it is not a resident of the grand regime. True grand-scale tail behavior is unknown, but because of possible divergences, due to incomplete theory, such unphysical divergence must be dealt with in grand-regime calculations.

We may express this explicitly by presuming that there exists a function that redistributes the probability additively to yield whatever the unknown but correct distribution may be, while preserving normalization [11]. This allows some flexibility in that we can begin with the thermalized wind case extended to three dimensions and add a redistribution function, R(v), which integrates to zero, to correct the PDF, i.e.,

(7) $$R\left(v\right)\equiv p_{\mathrm{correct}}\left(v\right)-p_{\mathrm{ref}}\left(v\right).$$

Here, $p_{\mathrm{correct}}\left(v\right)$ is the correct (normalized) PDF, and $p_{\mathrm{ref}}\left(v\right)$ is a normalized reference Gaussian that matches the core behavior of the correct PDF—see Equation (1) for the instance where *x* is v and thus, $x^{2}$ is ${\left|v\right|}^{2}$. An example is Equation (4) above, which, as we have seen, may result in divergence, forcing a recognition that true tail behavior must reflect population depletion.

Kinetic theory works from velocity moments of the PDF with constant factors that connect to the physical variables the integrals are meant to represent. Following [6], let $M_{n}\left\{g\left(v\right)\right\}\equiv \int_{D}\alpha_{n}\;g\left(v\right)\;d^{3}v$ be the velocity moments where the *n*th moment of g(v) results from the set $\alpha_{n}\in {\left\{1,v,|v\right|}^{2}\}$ for n=0,1,2, respectively. In kinetic theory, each $\alpha_{n}$ connects to a fundamental collisional invariant. The resulting moments may be scalars or vectors. In particular, $M_{1}\left\{g\left(v\right)\right\}$ is a vector. Then, we may compute $M_{n}\left\{p_{\mathrm{correct}}\left(v\right)\right\}$. Conditions on the zeroth and first moments arise, $M_{0}\left\{p_{\mathrm{correct}}\left(v\right)\right\}=1$ and $M_{1}\left\{p_{\mathrm{correct}}\left(v\right)\right\}=0$, because of normalization and symmetry, respectively.

Second moments are most relevant in connection with kinetic energy, K. K includes all components *i* of v. With *N* as the total number of particles in the entire system and *m* as the particle mass,

(8) $$\begin{array}{c} K=\frac{mN}{2}\sum_{i}M_{2}\left\{p_{\mathrm{correct}}\left(v\right)\right\}=\frac{mN}{2}\sum_{i}M_{2}\left\{p_{\mathrm{ref}}\left(v\right)\right\}+\frac{mN}{2}\sum_{i}M_{2}\left\{R\left(v\right)\right\}. \end{array}$$

The form of the first sum, involving $p_{\mathrm{ref}}$, follows naturally from standard integrals, so

(9) $$\begin{array}{c} K=\frac{3}{2}Nk\theta +\frac{mN}{2}\sum_{i}M_{2}\left\{R\left(v\right)\right\}. \end{array}$$

Recall here that classical temperature is not at play here. θ only arises because of the form presumed for $p_{\mathrm{ref}}$, and it includes thermalized winds.

In contrast, the second term in (9) is not generally known even if it exists because it depends on the function $p_{\mathrm{correct}}\left(v\right)$ relating the laboratory scale to the grand scale, and we have no experimental data (yet) to address how real PDFs become depleted at large |v|. The first two moments of the redistribution function must vanish, $M_{0}\left\{R\left(v\right)\right\}=0$ and $M_{1}\left\{R\left(v\right)\right\}=0$, the first by definition and the second because of symmetry.

If we define

(10) $$\varepsilon =\frac{mN}{2}\sum_{i}M_{2}\left\{R\left(v\right)\right\},$$

then (9) becomes

(11) $$K=\frac{3}{2}Nk\theta +\varepsilon .$$

We call ε *epitropy*, which is roughly analogous to entropy. However, entropy represents hidden properties of the laboratory regime. The term suggests hidden *inner* dynamics. In contrast, epitropy concerns hidden *outer* dynamics. This is a revision of the definition proposed in [6] to allow for a more general range of possible tail behaviors as discussed above. The first term in (11) connects K to more familiar forms in the laboratory regime in which *N* signals an extensive property. *N* is included in the definition of epitropy, making it extensive and preserving the analogy to entropy.

Since the redistribution function integrates to zero, it must both add and subtract to take the classical Gaussian to a properly depleted tail. One might expect that the resulting ε is positive for heavy tails and negative for slim tails. But this remains unclear, given that the PDFs for heavy tails must cross the Gaussian case in order to preserve normalization as seen in Figure 1.

## 4. Epitropy and Diffusion

Equation (11) is analogous to the traditional equation of state for an ideal gas, $E=\frac{3}{2}NkT$, which comes directly from kinetic integrals assuming a strictly Maxwellian distribution. In this section, we use these integrals to address grand-regime dynamics. First, we proceed to a continuum by allowing *N* to be a number density, N(r,v,t), instead of a total number. Then, we use classical kinetic integrals, but with the revised PDFs that account for convolution and tail depletion [11], resulting in the form (11), allowing the emergence of epitropy.

The mean occupation number is n(r,v,t), where n=N(r,v,t)p(r,v,t). Collisional invariants are *m*, the mass per particle, and $K=mv^{2}/2$, the kinetic energy per particle. Momentum is also such an invariant but the assumption of slow time in place so far is that the mean velocity of wind averages to zero. This is not a necessary condition, but it has proven convenient. However, that does not mean that the system is in full equilibrium. There can be gradients in the overall kinetic energy over r.

Applying the chain rule, the rate of change of n(r,v,t) is

(12) $$\frac{dn}{dt}=\frac{\partial n}{\partial t}+v\cdot \nabla n,$$

where we ignore body forces. This gives a Boltzmann-like equation if equated to a collision term

(13) $$\frac{\partial n}{\partial t}+v\cdot \nabla n=c.$$

*c* is a collision term. The collision term accounts for particle numbers that are removed and added to the velocity stream by collisions. As atomic collisions are treated as elastic, this means that at any point, (r,v), the resulting mass, momentum, and kinetic energy gain by collisions must be matched by an identical mass, momentum, and kinetic energy loss. Thus, the moments of collisional invariants of *c*, $\int \alpha_{n}\;c\;d^{3}v$, must all vanish [12].

The moments of the collisional invariants for (13) yield the key physical equations in kinetic theory [12],

(14) $$\int \alpha_{n}\frac{\partial n}{\partial t}d^{3}v+\int \alpha_{n}v\cdot \nabla n\;d^{3}v=\int \alpha_{n}\;c\;d^{3}v.$$

Taking care not to think that *v* depends on position, we extract an equation for each moment. As the average velocity vanishes, u=0, by assumption, each term for n=0, and n=1 will vanish, leaving only n=2 as a viable equation for dynamics. For n=2, we introduce the total kinetic energy in a volume, $K=\int Kn\;d^{3}v$, where *K* is the kinetic energy per particle. Using the result from moments above, we find

(15) $$\frac{\partial K}{\partial t}+\nabla \cdot J=0,$$

where J is the flux density of kinetic energy, $\int \frac{1}{2}mv^{2}nv\;d^{3}v=\int Knv\;d^{3}v$. The second form illustrates the meaning of a flux density in this case. That integrand shows the kinetic energy per particle times the fraction of particles with that energy carried with velocity v.

Equation (15) represents the conservation of energy in this simple environment. It has been unnecessary to specify *n* or *m*. Equation (15) is purely formal and simply indicates conservation generally. It is generic, applying broadly to any accountable quantity in a continuum without a source or sink. Applying it to the grand regime requires Equation (11).

J is more challenging in the grand-scale regime. If we employ the Fourier law J∝−∇K, we proceed easily but with the provision that one may not defer to experience to justify such a choice. However, it does make sense in that processes are rooted in differences and the only property that can have a difference to cause a flux are gradients in K. More imaginative alternatives may emerge, but we will not consider them here.

We thus find the standard diffusion equation,

(16) $$\begin{array}{c} \nabla^{2}K=D\frac{\partial K}{\partial t}, \end{array}$$

where *D* is a suitable diffusion constant. This looks like classical diffusion; however, it does not lend itself to an equation in simple temperature which the diffusion equation usually allows, because we only have the generalized temperature θ that includes thermalized Gaussian wind, and there is epitropy too.

Equation (16) is the standard diffusion equation. It represents how kinetic energy moves in time in this case. But we may use (11) to see how the classical-like term and epitropy move in the grand regime,

(17) $$\begin{array}{c} \frac{3}{2}Nk\nabla^{2}\theta +\nabla^{2}\varepsilon =D\frac{3}{2}Nk\frac{\partial \theta }{\partial t}+D\frac{\partial \varepsilon }{\partial t}. \end{array}$$

Of course, in the sub-Gaussian case, the epitropy terms may prove to be negligible, but for the super-Gaussian case, that need not be so. Thus, we may also consider a case where gradients in the generalized temperature are absent. In that case,

(18) $$\begin{array}{c} \nabla^{2}\varepsilon =D\frac{\partial \varepsilon }{\partial t}. \end{array}$$

It is worth recalling the chain of reasoning that took us to (18). Tail behavior in classical statistical mechanics and kinetic theory is sensibly dismissed as negligible. However, fluctuating local equilibrium can result in heavy-tail PDFs. Such a PDF in (14) leads to divergent integrals, which is aphysical. A proper outcome for (14) requires us to discard Maxwellian tails, and address how PDFs must become depopulated for large |v|. Epitropy, which we represent with ε, addresses this directly. Seemingly negligible features in the laboratory regime may emerge to drive processes in the grand regime.

## 5. Conclusions

*Temperature and PDFs:* Natural physical systems in the open are not in thermodynamic equilibrium, but are often usefully represented as being in local thermodynamic equilibrium. Furthermore, these local states are typically fluctuating in time. In this paper, we have focused on classical fluids, which have been the subject of much attention in kinetic theory, with the aim of linking the micro-regimes to the laboratory scales. In the classical regimes, the focus is on the Maxwellian velocity PDF, which gives classical temperature. Quantum alternatives exist, but we have not considered Bose–Einstein or Fermi–Dirac alternatives. All of these can be deduced from a constrained maximum entropy scheme wherein the Largrange multiplier of the constraint produces temperature.

*Kinetic theory and the Maxwellian:* The Maxwellian in a fluctuating medium works satisfactorily in the laboratory regime. Through kinetic theory, it provides the classical temperature of the laboratory regime. However, if one wishes to address long time and large space scales, the laboratory-scale fluctuations must be incorporated. The traditional approach is to take the approximate forms from the laboratory regime arising from the Maxwellian or nearly Maxwellian distributions via kinetic theory, and then average them in search of a grand regime.

*Incompleteness of classical fluids:* Averaging the emerging dynamical equations has never been successful except in an ad hoc manner. The failure is known as *the closure problem* of fluid mechanics. Kinetic theory arrives at its best representations of the laboratory regime by approximate methods such as, for example, the Chapman–Enskog expansion to solve the Boltzmann equation. This leads to highly complicated higher-order terms which are normally discarded. That means that classical fluid mechanics is incomplete in some sense. Does this incompleteness play into the closure problem? That question gives one strong motivation for the present work.

The classical kinetic theory does not prescribe or define specific length and timescales, or prescribe any sort of hierarchy of regimes. It takes a PDF (Maxwellian in the classical case), which then becomes the heart of an “averaged” regime through kinetic integrals. Different PDFs induce different physical regimes. To study the specifics of the particulars of the transition from one regime to the next requires more than the tools of kinetic theory. In kinetic theory, the results are always after the fact of transition. A sequence of PDFs leads to a sequence of regimes. If the PDF forms some sort of hierarchy, then one may say that the resulting regimes form a hierarchy in that sense.

*This paper:* We have taken a fundamentally different approach from the classical derivation of fluid mechanics by using these properties of PDFs instead of averaging the classical laboratory-scale equations. To capture long time and large space scales, we began by deducing a more appropriate PDF for such scales and then applied kinetic integrals to the results in this paper. While the effect of discarding higher-order terms in the classical approach is difficult to assess, the task of assessing what are appropriate PDFs is also challenging. To make some progress, we have limited ourselves to making some convenient but plausible assumptions.

If we begin with the Maxwellian, we observe that there are two ways for local fluctuations in equilibrium to manifest themselves: fluctuations in local wind velocity, and fluctuations in local temperature. However, if we wish to progress beyond these simple properties, we require some plausible assumptions. In the case of fluctuating wind speed, many possibilities exist. For example, it is known from time series analysis that climate statistics shift from being persistent on meteorological timescales to statistically anti-persistent on longer timescales [13]. This opens the prospect of non-Gaussian PDFs on very large timescales. Also, on short timescales, turbulent flow is notoriously non-Gaussian.

*Some Assumptions:* To proceed, we assumed that the fluctuations in velocities have the well-precedented Gaussian form for simplicity. Furthermore, we assumed that the resulting mean velocity canceled to zero. This assumption is not necessary. One could imagine persistent trade winds for example. The result is that wind becomes fully “thermalized” in that the PDF is fully Gaussian with simply a revised parameter that one might call temperature-like, θ. There is no wind in this version of the grand regime.

Complications arise, however, when we attempt to address temperature fluctuations. These lead to new non-Gaussian PDFs even when starting from Gaussian or truncated Gaussian fluctuations. This outcome is inevitable because temperature appears in the ground Maxwellian’s normalization factor, unlike velocity. The resulting PDFs have a Gaussian core with heavy (i.e., polynomial) tails. This outcome has some serious consequences. First, non-Gaussian forms in classical physics means that there is no temperature in the grand regime. This is not unprecedented as physical variables come and go across regimes. Second, the kinetic theory strategy becomes problematic as second moments will diverge.

*Divergence and Epitropy:* One might fix this by convolving with a temperature fluctuation PDF with clipped tails (i.e., box car PDF). This eliminates the heavy tail problem, but the result is sub-Gaussian (i.e., still no temperature). Furthermore, this fix is artificial as there is no way to know a priori on what grounds one should clip tails. This leads to a renewed scrutiny of the Maxwellian tails. In other contexts, this question never arises; however, divergent moment integrals bring this issue into focus here. Instead of clipping tails of the convolution, the tails of the classical PDF are naturally limited. There are only a finite number of particles and only a finite amount of energy for them, which means that the continuum assumption must always break down for large enough velocities. Formally, this means that real PDFs are always on a finite domain, not infinite or semi-infinite. But that introduces another issue. How the tails become depopulated can happen in many distinct ways. As the depletion prevents integral moments from diverging in certain circumstances, the specifics cannot be ignored. This leads to the notion of epitropy, which captures the hidden aspects of the depleted tails in the second moment integral by using the redistribution functions [11] against a reference Gaussian PDF.

*Diffusion of Epitropy in Grand-Regime Dynamics:* With a simple gradient assumption, the kinetic integrals complete the program arriving at the classical diffusion equation for energy. From that, one observes a special case wherein epitropy alone drives the movement of energy. This “tail wagging the dog” scenario demonstrates the physical reality of the depopulation of PDF tails for very long timescales and large space scales. The unseen grand regime is rooted in other features of nature that we do not see on our time and space scales (the laboratory regime).

Classical PDFs are presumed to have conveniently infinite domains. The search for dynamics native to large scales, however, leads to some divergent moment integrals. The natural remedy is to embrace finite domains, as both energy and particle number are actually finite. The formation of the finite domain introduces a new general physical consideration, which is dealt with by introducing the concept of epitropy. It is shown to be a natural part of dynamics on the grand scale. 

## Data Availability

All information is included in the paper. Inquiries may be directed to either of the authors.

## Abbreviation

The following abbreviation are used in this manuscript:

PDF	probability distribution function
